# *Lysinibacillus* Isolate MK212927: A Natural Producer of Allylamine Antifungal ‘Terbinafine’

**DOI:** 10.3390/molecules27010201

**Published:** 2021-12-29

**Authors:** Sayed E. El-Sayed, Neveen A. Abdelaziz, Hosam-Eldin Hussein Osman, Ghadir S. El-Housseiny, Ahmed E. Aleissawy, Khaled M. Aboshanab

**Affiliations:** 1 Department of Microbiology and Immunology, Faculty of Pharmacy, Ahram Canadian University (ACU), Sixth of October City 12451, Egypt; sayed.emad@acu.edu.eg (S.E.E.-S.); neveen.abdelaziz@acu.edu.eg (N.A.A.); 2 Department of Anatomy, College of Medicine, Taif University, P.O. Box 11099, Taif 21944, Saudi Arabia; h.hussein@tu.edu.sa; 3 Department of Microbiology and Immunology, Faculty of Pharmacy, Ain Shams University, Organization of African Unity St., Cairo 11566, Egypt; ghadir.elhossaieny@pharma.asu.edu.eg; 4 Department of Pharmacognosy, Faculty of Pharmacy, Ain Shams University, Organization of African Unity St., Cairo 11566, Egypt; aelissawy@pharma.asu.edu.eg

**Keywords:** antifungal, *Lysinibacillus*, terbinafine, bio-active metabolite, fungicidal, 2D NMR

## Abstract

Resistance to antifungal agents represents a major clinical challenge, leading to high morbidity and mortality rates, especially in immunocompromised patients. In this study, we screened soil bacterial isolates for the capability of producing metabolites with antifungal activities via the cross-streak and agar cup-plate methods. One isolate, coded S6, showed observable antifungal activity against *Candida (C.) albicans* ATCC 10231 and *Aspergillus (A.) niger* clinical isolate. This strain was identified using a combined approach of phenotypic and molecular techniques as *Lysinibacillus* sp. MK212927. The purified metabolite displayed fungicidal activity, reserved its activity in a relatively wide range of temperatures (up to 60 °C) and pH values (6–7.8) and was stable in the presence of various enzymes and detergents. As compared to fluconazole, miconazole and Lamisil, the minimum inhibitory concentration of the metabolite that showed 90% inhibition of the growth (MIC_90_) was equivalent to that of Lamisil, half of miconazole and one fourth of fluconazole. Using different spectroscopic techniques such as FTIR, UV spectroscopy, 1D NMR and 2D NMR techniques, the purified metabolite was identified as terbinafine, an allylamine antifungal agent. It is deemed necessary to note that this is the first report of terbinafine production by *Lysinibacillus* sp. MK212927, a fast-growing microbial source, with relatively high yield and that is subject to potential optimization for industrial production capabilities.

## 1. Introduction

High levels of resistance to antifungal agents have been recently reported particularly among pathogenic *Candida* spp. [1] and *Aspergillus* spp. isolates [2,3]. These elevated resistance rates are responsible for a largely silent epidemic and aggravated the prognosis of fungal infections, especially in the immunocompromised patients, leading to high morbidity and mortality rates [4]. *Candida* spp., for example, can cause a wide spectrum of systemic and invasive infections [5]. Invasive candidiasis was reported to cause about 20% of the serious illness in Intensive Care Units (ICUs) [6]. HIV-positive populations mainly suffer from oropharyngeal candidiasis as an opportunistic infection, with 95% of them showing infection by *C. albicans* [7,8,9].

Terbinafine hydrochloride, which is (E)-*N*-Methyl-*N*-(l-Naphtylmethyl)-6,6-dimethylhept-2-ene-4-ynyl-l-amine, was reported first in the European patent No.24587, as a good antifungal agent used preferably against mycosis caused by dermatophyte on the skin and on nails. This compound was prepared in three different ways, according to the process disclosed in this patent [10]. In a later publication [11], the hydrochloride salt of the trans isomer of terbinafine was prepared from the mixture of Cis and Trans isomers by column chromatography on silica gel and salt formation of base by treatment with hydrochloric acid in ethanol followed by recrystallization. Another study included the synthesis of chiral derivatives of terbinafine; a series of allylamine compounds have been synthesized and evaluated for their antifungal activity towards *Cryptococcus neoformans*. All compounds were chiral derivatives of terbinafine, having additional substituents at the carbon connected to the central nitrogen atom [12]. The stereo-selective synthesis of terbinafine, bearing (E)-tert-butylenyne structural element as the side chain, has been performed and resulted in a good yield. This methodology avoids the use of toxic starting materials [13]. A three-step batch-flow hybrid process has been developed for an expeditious synthesis of the enynol key intermediate of antifungal terbinafine. This procedure involves consecutive organometallic steps without the need for any in-line purification, enabling safe and convenient scale-up [14]. Therapies of fungal infections have been restricted by the limited numbers, low safety profiles and emergence of resistance to currently used antifungal agents [15]. No doubt we are facing a serious public health problem, which highlights the crucial need for the discovery of new specific antifungal agents, preferably from natural resources, particularly those that have not been exploited yet [16], for instance, soil microbial communities, which have been reported to produce biologically active natural byproducts [17,18,19,20]. This study is concerned with *Lysinibacillus* sp., a Gram-positive, rod-shaped bacterium from the family *Bacillaceae* [21]. These microorganisms were previously considered as members of the genus *Bacillus*, but due to the new taxonomic classification they now belong to the genus *Lysinibacillus* [22].

Bioactive metabolites derived from the genus *Bacillus* have showed structural diversity and a wide spectrum of bioactivities such as antifungal, antibacterial, antitumor and antimycoplastic activities [23]. The known *Bacillus* spp. that produce antifungal antibiotics are *B. subtilis*, *B. brevis*, *B. licheniformis*, *B. circulans* and *B. cereus* [24]. Most of peptide antifungal agents such as bacillomycin, mycobacillin and fungistatin are able to target and disintegrate fungal cell walls [25,26]. A recent study showed that fengycin and iturin can be used for biological control of *Mortierella* and *Myrothecium* and *Fusarium* [27]. Additionally, iturin can be used for the management of infections caused by *C. albicans* and several other human important fungal pathogens [28]. Both *B. subtilis* and *B. mojavensis* have a potential of lipopeptides and cell-wall degrading enzymes production, which act as an alternative resource for control of *C. albicans* [29]. Another 7-amino-acid residue lipopeptide antibiotic produced by *B. subtilis* named Surfactin showed antifungal activity against *Fusarium moniliforme* [30]. *Bacillus* strains (CWSI-B1567 and CWSI-B1568) also secreted antifungal metabolites that inhibited the growth of *C. albicans* [23]. Tyrocidine and Gramicidin S are cyclic peptides produced by *Brevibacillus brevis*. The gramicidin S synthetase, a multifunctional enzyme complex, catalyzes Gramicidin S synthesis [31]. *B. cereus* and *B. licheniformis*, which excrete several antifungal compounds, mainly lipopeptides, have also been successfully tested against candidiasis [32]. *B. cereus* produces cispentacin, which is active on *C. albicans* both in vitro and in vivo in mice, and mycocerein, an iturin antifungal, which inhibits growth [33]. Members of the *B. subtilis group* were found to produce numerous highly active antifungal compounds, e.g., Subtilin, subsporins A–C, lipooligopeptides, rhizocticins A-D and phosphooligopeptides [34]. Moreover, they produce lipopeptide bacillomycins L and D; bacillopeptins; and rhizocticin A, a hydrophilic phosphonooligopeptide active against *Candida* spp. [35].

Several metabolites produced by *Bacillus* sp. have been previously explored, but the antifungal potential of *Lysinibacillus* is poorly reported in literature. Therefore, this study aimed to identify the promising antifungal activity of a *Lysinibacillus* isolate recovered from soil against *A. niger* clinical isolate and *C. albicans* standard strain ATCC 10231. Subsequent purification, characterization and structure elucidation of the respective metabolite were performed, emphasizing the advantages of soil microbes as natural producers of bioactive metabolites. 

## 2. Results

### 2.1. Screening for the Production of Antifungal Metabolite(s)

Seventy-three bacterial colonies were isolated and screened using the cross-streak, agar cup-plate and dual-culture methods. Results showed that only three isolates (coded F1, S6 and F2) out of 73 (4.1%) isolates gave clear halos of inhibition against *C. albicans* ATCC 10231 and *A. niger* clinical isolate, indicating their antifungal activities (Figure S1). The isolate S6 showed maximum inhibition zone as compared to F1 and F2 and therefore was chosen for further study (Table S1).

### 2.2. Identification of the Isolate S6

**Figure 1:**
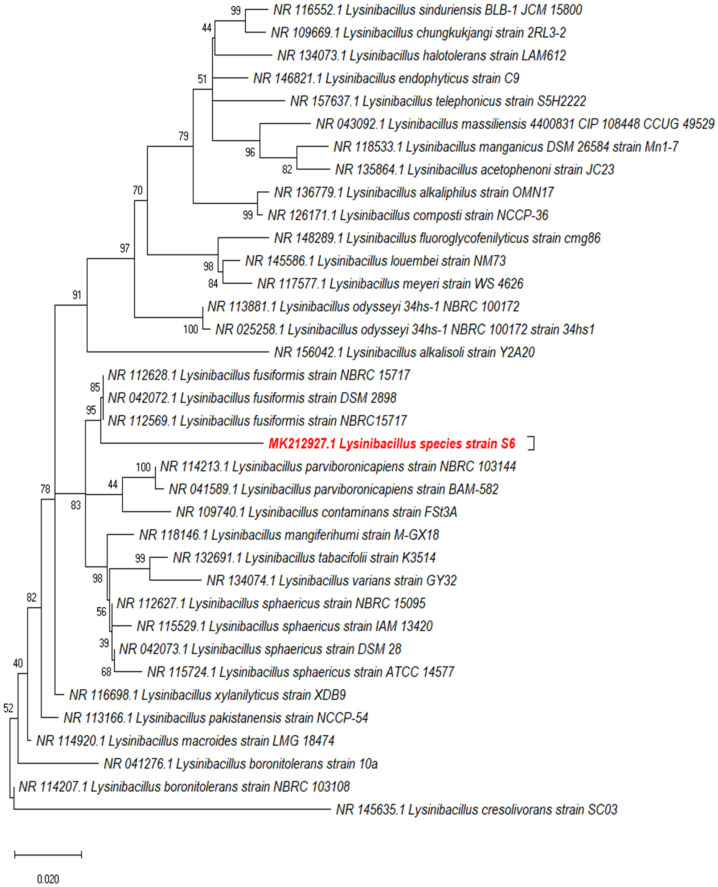
Molecular phylogenetic analysis of the query isolate (S6) using maximum likelihood method based on the Kimura 2-parameter model in MEGA X.

A phylogenetic tree of the 16S ribosomal RNA gene sequence was constructed using the MEGA X and showed that the S6 isolate clustered in close vicinity to *Lysinibacillus *(L.)* fusiformis* but occupied a separate sub-branch (Figure 1). Isolate S6 was spore-forming Gram positive rods, which gave positive for starch hydrolysis, catalase production and citrate utilization and negative for gelatin liquefaction, casein hydrolysis and urease production. According to the obtained results, isolate S6 was identified as *Lysinibacillus* isolate MK212927 (NCBI nucleotide accession code, MK212927.1) and deposited in the Culture Collection Ain Shams University CCASU-MK212927.

### 2.3. Extraction of the Antifungal Metabolite(s)

No precipitation of any peptides was seen in the culture supernatant using (NH₄)₂SO₄ at the stated conditions. Ethyl acetate was the optimum solvent for extraction, with a net weight yield of 1.07 g from 200 mL of clear supernatant, and resulted in inhibition zones of 26.6 mm and 24.3 mm against *C. albicans* standard strain and *A. niger* clinical isolate, respectively (Table S2).

### 2.4. Purification of the Bioactive Metabolite(s) by Silica Gel Column Chromatography

The solvent system consisting of hexane: ethyl acetate was used for extract fractionation (Table 1). About 136 fractions were collected and pooled into 21 pooled fractions (PFs) based on their TLC retardation factors. The fractions were checked by means of bioautography for their antifungal activities, where the PFs 6, 7 and 8 showed maximum antifungal activities (Figure S2). Other PFs showed either no or moderate activities. Elutes 43–47 forming the pooled fraction PF 6 showed the maximum antimycotic activity and were harvested using solvent system hexane: ethyl acetate with the ratio 75:25 (Table 1).

### 2.5. Correlation between the Metabolite(s) Concentration and Inhibition Zone Diameters Using Agar Well Diffusion

**Figure 2:**
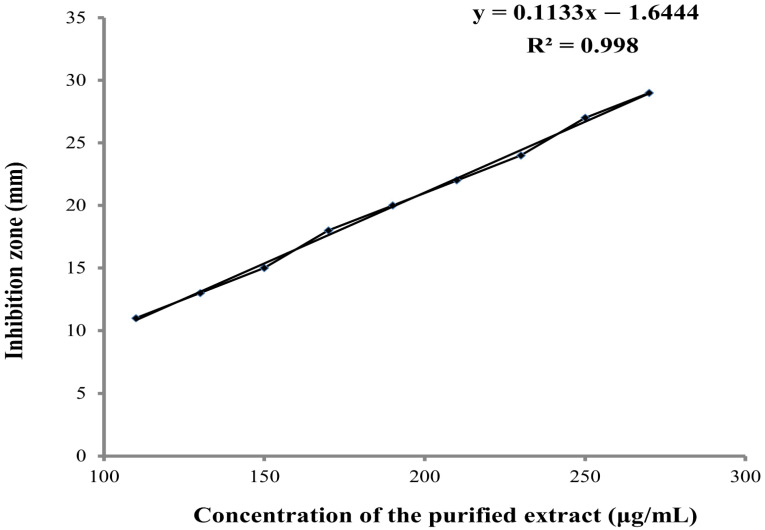
Linear relationship between different concentrations of the purified extract (µg/mL) and the inhibition zone diameter (mm).

As shown in Figure 2, the increase in the concentrations of the purified antifungal compound was associated with a linear increase in the inhibition zones (mm) of the respective concentration.

### 2.6. Thermal, Enzymes, Detergents, and pH Stability of the Antifungal Metabolite

The compound was found to be a light brown amorphous solid. It was soluble in most organic solvents. As shown in Figure S3, the bioactive metabolite retained its activity up to 60 °C; however, reduced activity was observed as the temperature exceeded 70 °C, and its activity was lost after autoclaving for 15 min at 121 °C. On the contrary, the antifungal activity was preserved in the presence of proteinase-K, lysozyme and alpha-amylase. Similarly, the antifungal activity was not significantly affected by the presence of tween 20, 40, 80 nor SDS and cetrimide (Figure S3). It also reserved its activity over a wide pH range (6.0–7.8) (Figure S3). Moreover, the antifungal compound produced by *Lysinibacillus* isolate MK212927 was assayed for fungistatic or fungicidal mode of action. The fungus *A. niger* showed no regrowth after it had been transferred to a new SDA plate and monitored over 7 days. These results indicated that the metabolite produced has fungicidal activity.

### 2.7. Evaluation of the Antifungal Activity of the Lysinibacillus Isolate MK212927 Metabolite Compared to the Commercially Available Antifungal Agents

**Figure 3:**
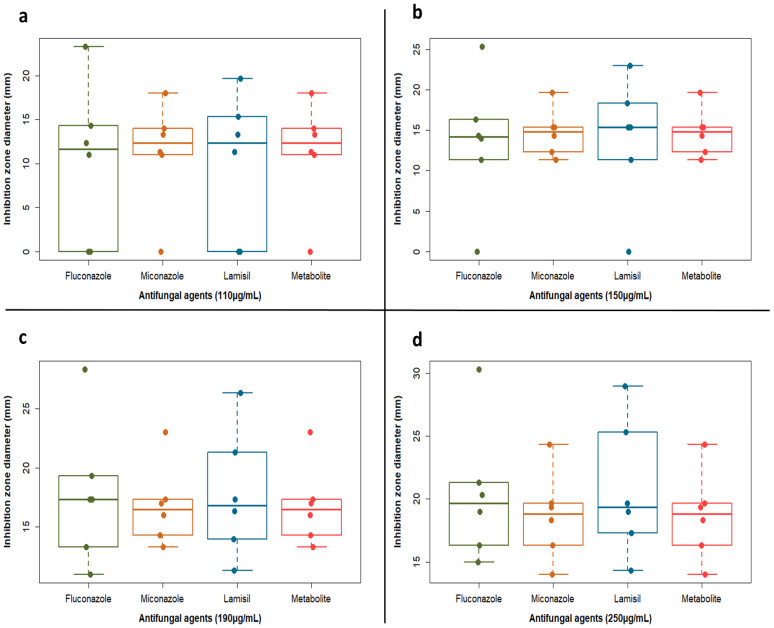
Boxplots showing inhibition zone diameter (mm) vs. antifungal agents (fluconazole, miconazole, Lamisil and the active metabolite) at different concentrations: (**a**) 110 µg/mL, (**b**) 150 µg/mL, (**c**) 190 µg/mL and (**d**) 250 µg/mL. Dots representing each sample were overlaid to show the actual variability and distribution of the data. Plot was created by R (“ggplot2”) package.

**Figure 4:**
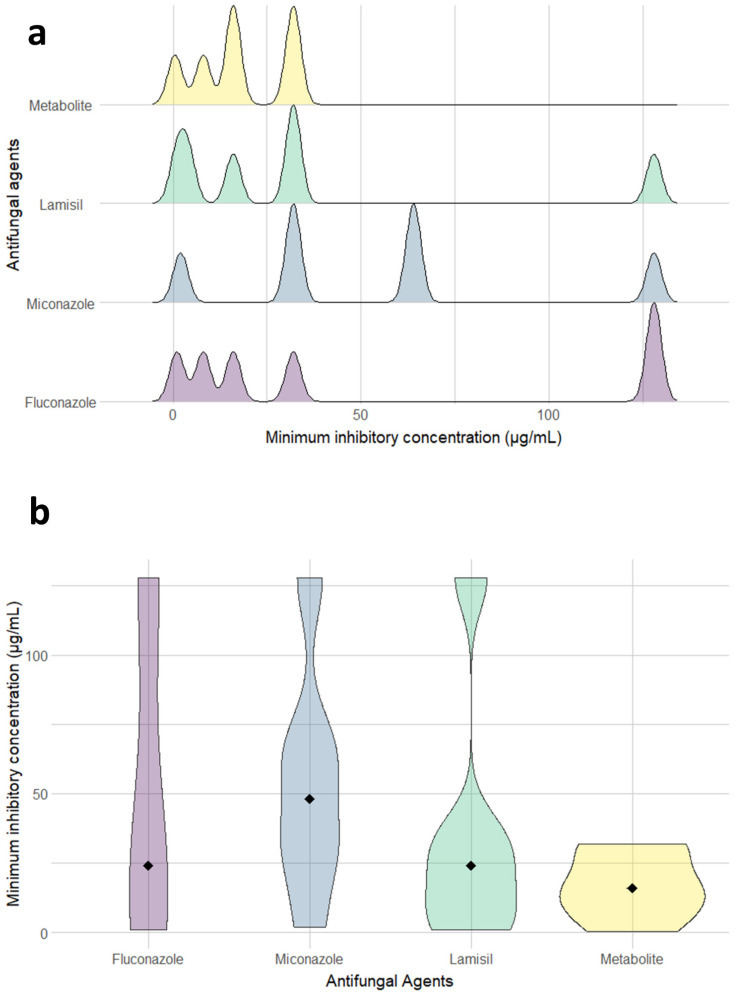
(**a**) Ridgeline plots showing the distribution of minimum inhibitory concentrations (µg/mL) for fluconazole, miconazole, Lamisil and the metabolite against six isolates of *C. albicans.* Area under the ridgeline is plotted after calculation of density estimates from the provided data. Plot was created by R (“gg ridges”) package. (**b**) Violin plots representing the kernel probability density of *C. albicans* isolates at different values of the minimum inhibitory concentrations (µg/mL) for fluconazole, miconazole, Lamisil and the metabolite. The filled black diamond signifies the MIC median value allowing better assessment of the data obtained from different antifungals. Plot was created by R (“ggplot2”) package.

Table 2 illustrates the antifungal spectrum of three chosen antifungal agents and the purified metabolite at concentrations of 110, 150, 190 and 250 μg/mL against *C. albicans* clinical isolates. The purified metabolite showed comparable results to the chemical agents, with inhibition zones in the range of 11–25 mm as against 11–30, 11–25 and 11–29 mm zones of fluconazole, miconazole and Lamisil, respectively. Mostly, inhibition zones documented for the metabolite were less dispersed around the median and with very few outliers (Figure 3). Interestingly, the purified compound showed enhanced antifungal activity (i.e., inhibition zones between 11 and 12.5mm) against isolates 1 and 3 exhibiting resistance to fluconazole (i.e., no inhibition zones) at concentrations of 110 and 150 μg/mL. It is worth mentioning that MIC determination using broth microdilution method confirmed the promising inhibitory activity of the metabolite with results in the range of 0.5–32 μg/mL, while fluconazole, miconazole and Lamisil showed an extended range of MIC values reaching 128 μg/mL (Figure 4a and Table S3).

The metabolite had a median MIC value of 16, which was one third that recorded by miconazole and slightly less than that recorded by Lamisil and fluconazole (Figure 4b and Table 3). Next, we used Kruskal–Wallis One Way Analysis of Variance to test the differences between the effects of the tested antifungal agents. Despite the trend of discrepancies in the recorded medians among the tested antifungals, these differences did not reach statistical significance (*p*-value = 0.427); this could be due to the low number of isolates in each group. However, the metabolite displayed MIC_90_ 32 μg/mL, which was equipotent to Lamisil, 2-fold more potent than MN and 4-fold the potency of fluconazole (Table 3). Of note, the results also emphasized the improved activity against the two isolates displaying resistance to fluconazole, miconazole and Lamisil according to the interpretive guidelines of CLSI for in vitro susceptibility of *Candida* spp.

### 2.8. Spectral Analyses of the Purified Antifungal Compound

The ultraviolet (UV) absorption spectrum showed a maximum absorption peak (λ max) at 219 nm (Figure S4). The FTIR chart showed no absorption peaks between 3100 and 3500 cm^−1^ nor between 1630 and 1820 cm^−1,^ which indicated that the compound had no OH stretch nor carbonyl groups, respectively. On the other hand it exhibited an absorption at 3000–3100 cm^−1^ characteristic of the (SP2-1s)=C-H bonds in aromatic rings. In addition, aromatic rings showed absorption in the region 690–900 cm^−1^ because of the out plane C-H bending. Besides, the compound showed several absorptions between 1450 and 1600 cm^−1^ owing to C=C stretching in the aromatic ring. In the double bond region, a strong peak at 1600 cm^−1^ denoted the presence of an alkene (Figure S4). The obtained results were in accordance with those of the standard terbinafine (Figure S4).

**Figure 5:**
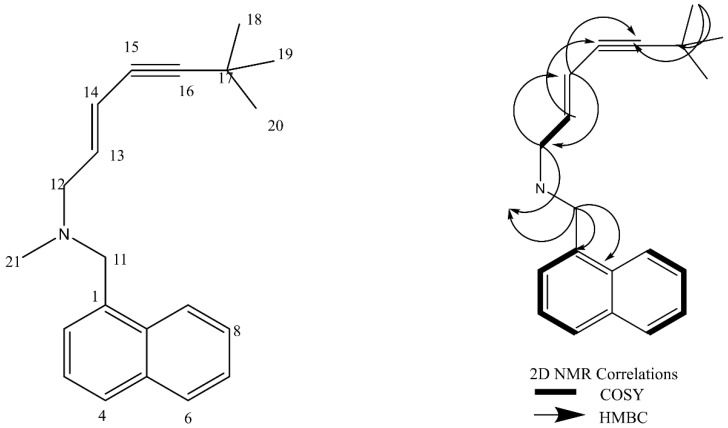
Chemical structure of the active metabolite produced by *Lysinibacillus* isolate MK212927 based on the spectral data showing ^1^H NMR,^13^C NMR signals assignments and 2D NMR (COSY, HMBC) correlations.

Results of extensive 1D and 2D NMR experiments are listed in Table 4. The chemical structure of the active metabolite produced by *Lysinibacillus* isolate MK212927 based on the spectral data showing ^1^H NMR,^13^C NMR signals assignments and 2D NMR (COSY, HMBC) correlations is delineated in Figure 5. Careful analysis of the generated spectra (Figures S5 and S6) showed great resemblance to those reported in the literature for the potent antifungal drug terbinafine (Lamisil)^®^ [36]. Terbinafine is a synthetic allylamine; however, we report a new microbial producer of this highly active antifungal compound.

## 3. Discussion

In this study, a total of 105 soil samples were gathered from various regions in Egypt and were processed for the isolation of bacterial strains showing inhibitory effect against two clinically relevant human fungal pathogens. Cross-streak and agar cup-plate methods were used to verify the production of the antifungal metabolite. Both methods confirmed that isolate S6 strongly inhibited the growth of fungal target pathogens. This Gram-positive isolate was identified as *Lysinibacillus* sp. MK212927 based on biochemical tests and 16S ribosomal RNA sequence (NCBI GenBank accession number: MK212927.1). Previous studies reported that the genus *Lysinibacillus* can produce valuable bioactive agents [37,38,39], while Ahmad and colleagues [40] described the antibacterial nature of the cell-free supernatant of a certain *Lysinibacillus* isolate. In another study, the species *Lysinibacillus* *fusiformis* were found to produce potential antifungal metabolites against certain fungi causing plant diseases [41]. However, comparison of the obtained cultural, biochemical and molecular characteristics, from the nucleotide DNA sequences of the 16S ribosomal RNA of our *Lysinibacillus* isolate MK212927 to those of the related *Lysinibacillus* sp. [22], indicated that our isolate was different from these previously reported *Lysinibacillus* spp. This species difference was also confirmed by using molecular phylogenetic analysis of our isolate using maximum likelihood method based on the Kimura 2-parameter model in MEGA X software. Our data supported these studies, demonstrating the potent antifungal properties of the metabolite produced by our *Lysinibacillus* isolate MK212927.1. Additionally, we have described for the first time the significant inhibitory effect of this antifungal metabolite against clinical isolates of the human pathogenic fungi *C. albicans* and *A. niger.* The optimized factors affecting the production of this metabolite were reported in our previous study [42].

The present investigation correspondingly defines the nature of the metabolite produced by *Lysinibacillus* isolate to elucidate its structure and demonstrate the effect of different factors on its activity. As a start, tests performed indicated that the anti-fungal metabolite was not proteinaceous in nature. Eight organic solvents were used to extract the active metabolite. The maximum yield (1.07 g/200 mL) was obtained by using ethyl acetate. This is in accordance with many reports where most of the antifungal metabolites are extracted with ethyl acetate [43,44]. Next, we purified the compound from the culture extract of *Lysinibacillus* isolate MK212927.1 using silica gel column chromatography techniques. Silica gel could tightly attach with high polar compound; therefore, low polar compounds were eluted from column before high polar compounds, which were sequentially eluted by increasing polarity of developing solvent [45]. Fractions 43–47 were the most bioactive ones, with confirmed antifungal activity verified by bioautography against *C. albicans.* A linear relationship was observed between the inhibition zones and the concentration of the purified extract; both increased proportionally.

Generally, secondary bacterial metabolites are stable because they are end products and not subject to alteration by the producing microorganisms. On that account, we subjected our bioactive metabolite to stability studies by examining the influence of various factors (temperature, pH, detergents and enzymes) on its antifungal activity. The metabolite was moderately thermostable, with the highest activity recorded at 30 and 40 °C, but totally lost its activity when autoclaved. It retained its maximum activity around neutral pH; this is in accordance with the properties of a previously reported antifungal metabolite produced by *Bacillus* spp. [46]. Moreover, the metabolite reserved its antifungal activity regardless of the employed enzymes and detergents, which highlights advantageous features at the industrial level.

Next, we compared the activity of commonly used antifungals to the purified metabolite. Intriguingly, the metabolite exhibited analogous activity to all the chemical agents and even superior outcome in some cases. In comparison to the tested antifungal agents, the minimum inhibitory concentration of the antifungal compound that inhibited 90% of the growth (MIC_90_) was equivalent to that of Lamisil, half of miconazole and one fourth of fluconazole. Even though fluconazole is the most common treatment of *Candida* spp. infections, resistant strains have emerged due to its repeated use [47]. An additional advantage of the metabolite was the enhanced effect against *C. albicans* clinical isolates resistant to the tested agents, fluconazole, miconazole and Lamisil. Antifungal compounds usually exhibit either a fungistatic or fungicidal mode of action in preventing fungal growth. Fungicidal compounds kill fungal cells, hence, no rejuvenation occurs after the compound is removed. On the contrary, fungistatic agents are characterized by reversible growth inhibition, where fungal cells continue to grow upon elimination of the antifungal agent [48]. In this study, plating of the fungi taken from the dual assay plate did not show any resumed growth. This indicated fungicidal effect of the compound produced by *Lysinibacillus* isolate MK212927.1. Furthermore, meta-analysis studies on the pharmacotherapy of invasive candidiasis detected higher therapeutic success rates among patients treated with fungicidal therapy when compared to fungistatic therapy [49,50]. Spectral analyses, including IR, 1D-NMR and 2D-NMR experiments (COSY, HSQC and HMBC), have been used in attempt to elucidate the structure of the compound. The data provided by these analyses confirmed the structure to be identical with that of terbinafine [51,52].

Terbinafine was discovered in 1983 and approved in 1996 in the US as the first oral allylamine antifungal agent [53]. Terbinafine inhibits ergosterol synthesis, an essential component of the fungal cytoplasmic membrane, by inhibiting the fungal cell membrane synthesis pathway through inhibition of squalene monooxygenase (squalene 2, 3-epoxidase) enzyme [54]. Since its approval, terbinafine has demonstrated high cure rates and well-established safety record. In contrast to azoles, terbinafine does not inhibit CYP3A4 isoenzyme during its metabolism and thus drug–drug interactions are low. This advantage enables the use of terbinafine for patients who are likely to be receiving concomitant medication, including antidiabetic and antihypertensive drugs [55]. Terbinafine is synthetically developed by Novartis under the trade name Lamisil^®^. Hence, the significance of this study remains that it reports the use of a new microbial process to produce terbinafine. Drugs produced by microbial processes surpass chemically produced ones in terms of their high specificity range and being easily produced from renewable carbon sources [56]. Recently, Abdel-Kader and Muharram obtained terbinafine from the culture media of *Streptomyces* spp. KHF12 isolated from a soil sample collected from Adilamm, KSA [57]. In our study, we used *Lysinibacillus* spp., a bacterium that passes through exponential phase during 3 to 10 h of growth and enters into stationary phase after 12 h of incubation, while typical *Streptomyces* colonies can be selected after 7 days [58]. Thus, our strain shows enhanced kinetics of terbinafine production that could be further optimized for industrial purposes.

## 4. Materials and Methods

### 4.1. Soil Sampling and Isolation of Bacterial Isolates

A total of 105 samples from soil were randomly gathered from various agricultural regions in Egypt over six months. An open-end soil borer was used to take samples from a depth of 10–20 cm then placed in tightly closed polyethylene plastic bags and stored at 4 °C for further processing. Briefly, 1 g of each soil sample was transferred into 100-mL normal saline, heated for 30–60 min at 80–100 °C to eliminate the non-spore-forming bacteria. Samples were then allowed to settle, and 100 μL of the supernatant was inoculated on yeast malt extract agar, starch casein agar, nutrient agar, trypticase soy agar and Sabouraud’s dextrose agar (SDA) and incubated for 1 week at temperatures of 28 °C and 37 °C [59]. The isolated colonies were then picked up, propagated on the same culture media, and preserved at 4 °C for later assays.

### 4.2. Screening for Production of Metabolites with Antifungal Activity

For the detection of diffusible antifungal metabolites, cross-streak method was used against *C. albicans* ATCC 10231. The screened isolate was streaked as a dense line in the center of the agar plate, and the *C. albicans* strain was streaked at right angle near the periphery of the plate. After incubation at 28 °C for 48 h, we recorded the size of inhibition zones [58,60]. Screening for the antifungal activity was also carried out using the dual-culture method against the mold *A. niger* clinical isolate recovered from Kasr Al-Ainy Hospital, Cairo university, Egypt (the ethical committee Nr. ENERC-ASU-230 at faculty of pharmacy and the hospital Ethics Committee have approved the study). Study protocols and experiments included agreed with international declarations for biomedical research ethics, relevant regulations, and guidelines. First, *A. niger* clinical isolate was cultured on SDA medium form 5 days to 1 week at 28 °C; then, a small block from the proliferated culture was centrally placed on another SDA plate. Then, a loopful of 24-h-old, screened isolate was cultured as a wide band approximately 3 cm away from the block of mycelia at two opposite margins. Plates cultured with fungus without any bacteria were served as control [61]. Finally, after 1 week incubation at 28 °C, the inhibition of fungal growth was calculated as the zone between the margins of streaked bacteria and the mycelia of the fungus [62].

$$\mathrm{Inhibition}\;(\%)=\frac{\mathrm{Radial}\;\mathrm{mycelial}\;\mathrm{growth}\;\mathrm{of}\;\mathrm{fungus}\;\mathrm{in}\;(\mathrm{Control}-\mathrm{Treatment})\;\mathrm{plate}}{\mathrm{Radial}\;\mathrm{mycelial}\;\mathrm{growth}\;\mathrm{of}\;\mathrm{fungus}\;\mathrm{in}\;\mathrm{Control}\;\mathrm{plate}}\times \;100$$

Isolates that showed preliminary antifungal activity were selected for confirmatory testing by the agar cup-plate method [63]. In brief, the isolates were inoculated in 100 mL of culture media under shaking conditions at 37 °C. After 24 h, the resulting culture broths were filtered then centrifuged at 6000 rpm for 15 min (Hettich Zentrifugen EBA 12, Tuttlingen, Germany) and the supernatants were examined for their antimycotic activity against *C. albicans* standard strain ATCC 10,231 (OD=0.5 McFarland standard at 530 nm). The plates were incubated at 28 °C, and the inhibition zones were measured after 24 h. Each experiment was performed in triplicate, and mean values of the inhibition zones diameters were recorded [64,65,66].

Ultimately, the bacterial isolate with the highest antimycotic activity was inoculated into TSB and incubated under shaking conditions (250 rpm) overnight at 37 °C. After that, centrifugation of 1 mL aliquots of these cultures was carried for 10 min at 10.000 rpm using a micro centrifuge (Hettich Zentrifugen EBA 12, Tuttlingen, Germany) in Eppendorf tubes. The obtained bacterial pellets were resuspended into 1 mL aliquots of glycerol stock medium. The Eppendorf tubes were then kept at −80 °C for long-term preservation of bacteria.

### 4.3. Identification of the Selected Isolate

The isolate showing the highest antifungal activity was identified using microscopical, biochemical and molecular approaches; 16S ribosomal RNA gene amplification and sequencing [67]. Staden package program was used to perform DNA analysis and sequence assembly (http://staden.sourceforge.net/ (accessed on 30 November 2021). The bioedit 7.2 software was used to get a consensus sequence of the 16S ribosomal RNA (https://bioedit.software.informer.com/7.2/ (accessed on 30 November 2021)), and by using BLASTn (Basic Local Alignment Search Tool of nucleotides) it was blasted in the GenBank database (http://blast.ncbi.nlm.nih.gov/Blast.cgi (accessed on 30 November 2021)). The homology percentage between the submitted sequence and the sequence in the data base was demonstrated. MEGA X software served to get the evolutionary analyses [68]. Alignment of the 16S rRNA sequence of each strain was done using MUSCLE against representative nucleotide sequences obtained from GenBank. Moreover, phylogenetic analyses were carried out through likelihood method based on the Kimura 2-parameter model with 1000 replicates bootstrap analysis [69]. The 16S ribosomal RNA sequence was deposited in the NCBI (accession number, MK212927), and the bacterial strain was deposited in the Culture Collection Ain Shams University (CCASU) belonging to WDCM (the World Data Centre for Microorganisms) (strain number, CCASU-MK212927).

### 4.4. Production of the Antifungal Metabolite(s)

A loopful from the selected bacterial culture (24 h old) was transferred to 50 mL trypticase soy broth (TSB) to prepare the seed culture and incubated for 15–18 h at 30 °C under shaking conditions (250 rpm). About 1 mL of the obtained culture was withdrawn to a micro centrifuge tube and then underwent centrifugation for at 16,000 rpm for 10 min (Centurion Scientific-K240R), rinsed with 1 mL sterile normal saline, and then transferred to 30 mL of the basal production medium. The basal production medium was prepared according to Singh et al. [41]. After inoculation by 1 mL of seed culture (final count of 1 × 10^7^ cfu/mL), the production flasks were incubated at 150 rpm and 30 °C.

### 4.5. Extraction of the Antifungal Metabolite(s)

The resulting culture broth from the production process was centrifuged for 30 min at 10,000; then, the resulting supernatant was harvested, filtered and stored at 4 °C for further assay [43,45]. Initially, to inspect peptide nature of the metabolite, (NH_4_)_2_SO_4_ (608 g/L) was added to the culture supernatant, kept overnight at 4 °C under shaking conditions, and monitored for peptide precipitation, if any [41].

For the extraction process, equal volumes of different solvents such as ethyl acetate (EA), chloroform, methanol, n-butanol, hexane, ethanol and diethyl ether were used [70]. Afterwards, the obtained extract was concentrated to dryness under reduced pressure at 45 °C using rotary evaporator (Staurt RE300, Warwickshire, UK) [45]. The antimycotic activities of the extracts were tested by using agar cup-plate method, and negative controls were prepared using the respective solvents [70,71]. The organic solvent that gave superior extraction evidenced by enhanced zone of inhibition was selected for further extraction procedures [43].

### 4.6. Thin Layer Chromatography (TLC) Analysis

TLC was the method of choice for the separation of the bioactive compound into its components. The crude EA extract was spotted on silica TLC plate and developed in different solvent systems: EA 1:9 hexane (*v*/*v*), EA 1:9 diethyl ether (*v*/*v*), methanol 1:9 EA (*v*/*v*), EA 1:9 chloroform (*v*/*v*), n-butanol 1:9 EA (*v*/*v*) and EA 1:9 dichloromethane (*v*/*v*). The separated metabolite(s) were then examined under UV lamp (UVitec^®^) at 254 nm and 365 nm and confirmed by the formation of localized brown spots after subjecting the TLC plates to iodine vapors in a fuming hood [45]. The solvent system that showed improved resolution of the spots was used at different ratios for the gradient elution of the compound.

### 4.7. Purification of the Bioactive Metabolite(s) through Activity Guided Fractionation

The dry mass of EA crude extract was adsorbed on a small amount of column grade silica gel, 60–120 mesh size (Merck, Germany). The silica gel column (3.5 × 80 cm) was first eluted with 100% n-hexane, and then we used linear gradients of n-hexane and ethyl acetate, increasing the polarity of the solvents. Fractions (each of 15 mL) were collected at regular intervals (1 mL/min flow rate) [70]. The fractions were loaded separately on silica TLC plates, developed with the same solvent system, and those exhibiting the same retardation factor (RF) values were pooled together [45]. The pooled fractions (PF) were concentrated using rotary evaporator at 45 °C; then, bioautography method was used to check their antifungal activity against test organism *C. albicans* ATCC 10231 [45]. The inhibitory zone resulting from bioautography method was visible after incubation at 28 °C for 1 day [72]. Fractions showing significant and potent antimycotic activity were re-purified as mentioned before; then, TLC plates were used to check their purity and were designated for further characterization [73].

### 4.8. Correlation between the Metabolite Concentration and Diameter of Inhibition Zone Using Agar Well Diffusion

The most active purified fraction of the metabolite obtained above was diluted in DMSO to achieve concentrations of 110, 130, 150, 170, 190, 210, 230, 250 and 270 μg/mL, and 200 µL of each concentration were added in separate cups in SDA plates inoculated with *C. albicans* ATCC 10231. After 24 h incubation at 28 °C for, the diameters of the inhibition zones were recorded and plotted against the corresponding concentrations.

### 4.9. Effect of Heat, pH, Detergents and Enzymes on Stability and Activity of the Antifungal Compound

To demonstrate the stability of the antifungal metabolite at different temperatures, 7 screw capped ampoules, each with 100 μg/mL of the metabolite, were kept in a water bath at temperatures 30, 40, 50, 60, 70 and 80 °C for 1 h, and the last ampoule was subjected to autoclaving temperature (121 °C for 15 min) [73]. Correspondingly, the effect of pH on stability of the metabolite was concluded through mixing 100 μg of the compound with 1 mL of 0.1 M phosphate buffer of different pH values (5.7–8.0) and then incubated at 30 °C for 1 h. Susceptibility of the antifungal metabolite to denaturation by various detergents, including tween 20, tween 40, tween 80, sodium dodecyl sulphate (SDS) and cetrimide, was also tested. Detergents were dissolved in distilled water at concentration of 0.01 g/mL. One hundred µL of the antifungal solution (100 μg/mL) was mixed with 10 mL of detergent and incubated at 30 °C for 6 h. The antifungal solution without any detergents served as control [74]. Similarly, we explored the effect of proteinase K, lysozyme and alpha amylase (Sigma-Aldrich, Cairo, Egypt) on the metabolite. All the enzymes were dissolved in distilled water at concentration 1 mg/mL, and 100 μL of the antifungal solution was mixed with 10 mL enzyme and incubated at 30 °C for 3 h. The antifungal solution without any enzymes acted as control [74]. The residual antifungal activities of the metabolite after heat, pH, detergents and enzyme treatments were determined by the agar cup-plate method using 100 µL of the antifungal compound per well [73]. Each experiment was performed in triplicate, and the mean values of the inhibition zones were recorded.

### 4.10. Investigation for Fungicidal and/or Fungistatic Mode of Action

A single 10-mm culture plug was taken from the edges of the inhibition zones of the *Lysinibacillus* isolate against the fungus *A. niger* and transferred to a fresh SDA plate, which was consequently incubated for a week at 28 °C [75,76]. Throughout the incubation period, growth or lack of growth of *A. niger* was examined. Regrowth was suggestive of fungistatic activity and absence of growth denoted fungicidal properties of the metabolite produced by *Lysinibacillus* isolate.

### 4.11. Evaluation of the Antifungal Activity of the Metabolite

We compared the efficacy of the metabolite to three antifungal products commonly used for treatment of *Candida* spp. infections, including fluconazole (Epico Pharmaceuticals Co., Ramadan City, Egypt), miconazole (Medical Union Pharmaceuticals, Nasr City, Egypt) and Lamisil (Novartis Pharmaceuticals, Cairo, Egypt). All tested agents were prepared at concentrations of 110, 150, 190 and 250 µg/mL in DMSO, and the respective solvent alone was used as control in the agar well diffusion method. We employed six clinical isolates of *C. albicans* with variable susceptibility profiles as our target organisms. The reported results are the mean values of three replicates ± standard deviation.

### 4.12. Minimal Inhibitory Concentration (MIC) Determination

The MIC values for the purified metabolite, fluconazole, miconazole and Lamisil were determined using different concentrations (0.125, 0.25, 0.5, 1, 2, 4, 8, 16, 32, 64 and 128 µg/mL) and following the Clinical and Laboratory Standards Institute (CLSI, 2018) protocol M27-A3 for fungi [77].

### 4.13. Spectral Analyses of the Antifungal Compound Assay

Ultraviolet (UV) spectrum was recorded on Shimadzu UV-1800 spectrophotometer, 1mg of sample was dissolved in 10 mL dimethylsulfoxide (DMSO) and the spectra were recorded at 200–400 nm range. Fourier transform infrared spectrum (FTIR) was determined by Bruker^®^ Optik GmbH, Germany in the range 400–4000 cm^−1^ by using methanol as the medium for the preparation of the sample. ^1^H NMR and ^13^C NMR spectra were measured in methanol on Bruker^®^ Avance III HD (400 MHz) spectrometer, Germany, equipped with a 5mm broad-band multinuclear (PABBO) probe. The chemical shifts were reported in parts per million (ppm) relative to TMS (δ0.0) used as internal standard. Two-dimensional (2D) NMR analyses, ^1^H-^1^H Correlation Spectroscopy (COSY), heteronuclear single quantum coherence (HSQC) and ^1^H-^13^C heteronuclear Multiple Bond Correlation Spectroscopy (HMBC) were also carried out on Bruker^®^ Avance III HD (400 MHz) spectrometer. All spectroscopic measurements were performed at Drug Discovery and Development Research Center at Faculty of Pharmacy, Ain Shams University, Abbassia, Cairo, Egypt.

### 4.14. Statistical Analysis

All experiments were carried out in triplicate, and the data were expressed as the mean ± SD. Statistical analysis was conducted using R statistical platform (https://www.r-project.org (accessed on 30 November 2021)). Among R-packages used in data analysis and visualization are readxl, ggridges and boxplot. Kruskal–Wallis one-way analysis of variance on ranks was used to test the significance among different groups. *p*-value < 0.05 was considered to indicate statistically significant differences.

## 5. Conclusions

In this study, we describe the isolation and purification of terbinafine from a new fast-growing microbial source, *Lysinibacillus* sp. MK212927, recovered from Egyptian soil. This highlights the role of microbial communities recovered from soil as an abundant resource of antifungal agents required to overcome the currently emerging fungal resistance. It is deemed necessary to note that this is the first report of terbinafine production by *Lysinibacillus* sp. MK212927, a fast-growing microbial source, with relatively high yield and subject to potential optimization for industrial production capabilities.

## Figures and Tables

**Table 1:** Fractionation of crude antifungal metabolite by *Lysinibacillus* isolate showing ratios of the solvent systems used, the retention factor(s), dry weight(s) and bioautography results.

Pooled Fractions (PFs)	n-Hexane: Ethyl Acetate (EA)	Elutes Recovered	Retardation Factor (R_F_)	Dry Weight of Each PF (mg)	Mean Inhibition Zones (mm) ± SD	
					*C. albicans*	*A. niger*
1	n-hexane 100%	1–7	0.96	1.4	-	-
2	95:5	8–19	0.93	1.8	-	-
3	90:10	20–25	0.88	4.6	16 ± 0.25	14 ± 0.25
4	85:15	26–36	0.84	2	21 ± 0.45	20 ± 0.36
5	80:20	37–42	0.78	3.33	22 ± 0.36	14 ± 0.25
6	75:25	43–47	0.71	4.1	31 ± 0.58	29 ± 0.58
7	70:30	48–53	0.69	2.4	30 ± 0.36	27 ± 0.36
8	65:35	54–59	0.63	4.22	29 ± 0.58	28 ± 0.58
9	60:40	60–64	0.60	2.6	13 ± 0.25	15 ± 0.36
10	55:45	65–69	0.58	0.99	-	-
11	50:50	70–76	0.52	0.89	-	-
12	45:55	77–82	0.49	4	-	-
13	40:60	83–88	0.46	3.9	-	-
14	35:65	89–94	0.43	3.7	17 ± 0.45	19 ± 0.58
15	30:70	95–99	0.39	1.67	18 ± 0.45	15 ± 0.25
16	25:75	100–106	0.35	2.78	14 ± 0.36	16 ± 0.36
17	20:80	107–112	0.31	4.4	12 ± 0.25	11 ± 0.25
18	15:85	113–119	0.28	1.8	15 ± 0.45	17 ± 0.36
19	10:90	120–125	0.23	1.01	20 ± 0.36	18 ± 0.36
20	5:95	126–130	0.14	5.2	19 ± 0.45	20 ± 0.58
21	EA 100%	131–136	0.12	4.9	23 ± 0.58	21 ± 0.36

**Table 2:** In vitro susceptibilities of six *C. albicans* isolates to fluconazole, miconazole, Lamisil, and the antifungal metabolite determined by agar well diffusion method.

*C. albicans* Isolate	Inhibition Zone Diameter (mm) ± SD			
	110 µg/mL	150 µg/mL	190 µg/mL	250 µg/mL
Fluconazole				
1	0	0	11 ± 0.33	15 ± 0.26
2	12 ± 0.58	14 ± 0.26	17 ± 0.33	20 ± 0.33
3	0	11 ± 0.33	13 ± 0.33	16 ± 0.58
4	11 ± 0.25	14 ± 0.25	17 ± 0.58	19 ± 0.33
5	23 ± 0.58	25 ± 0.58	28 ± 0.58	30 ± 0.33
6	14 ± 0.58	16 ± 0.58	19 ± 0.58	21 ± 0.58
Miconazole				
1	0	11 ± 0.33	14 ± 0.33	16 ± 0.33
2	13 ± 0.33	15 ± 0.33	17 ± 0.33	19 ± 0.67
3	11 ± 0.33	12 ± 0.46	13 ± 0.33	14 ± 0.26
4	11. ± 0.33	14 ± 0.36	16 ± 0.33	18 ± 0.33
5	18 ± 0.33	19 ± 0.67	23 ± 0.26	24 ± 0.33
6	14 ± 0.46	15. ± 0.33	17 ± 0.33	19 ± 0.33
Lamisil				
1	0	11 ± 0.33	14 ± 0.26	17 ± 0.33
2	11 ± 0.33	15 ± 0.33	16 ± 0.58	19. ± 0.67
3	0	0	11 ± 0.33	14 ± 0.58
4	15 ± 0.33	18 ± 0.5	21 ± 0.33	25 ± 0.58
5	19 ± 0.67	23 ± 0.33	26 ± 0.33	29 ± 0.6
6	13 ± 0.33	15 ± 0.33	17 ± 0.33	19 ± 0.6
Antifungal metabolite				
1	0	11 ± 0.33	14 ± 0.33	16 ± 0.33
2	13 ± 0.33	15 ± 0.66	17 ± 0.33	19 ± 0.69
3	11 ± 0.33	12 ± 0.33	13 ± 0.33	14 ± 0.45
4	11 ± 0.33	14 ± 0.33	16 ± 0.2	18 ± 0.33
5	18 ± 0.6	19 ± 0.67	23 ± 0.3	24 ± 0.33
6	14 ± 0.6	15 ± 0.33	17 ± 0.3	19 ± 0.67

**Table 3:** In vitro susceptibilities of six *C. albicans* isolates to fluconazole, miconazole, Lamisil, and the antifungal metabolite determined by broth microdilution.

Antifungal Product	Susceptibility (μg/mL)			
	Range	Median ^c^	MIC_50_^a^	MIC_90_^b^
Fluconazole	1–128	24	16	128
Miconazole	2–128	48	32	64
Lamisil	1–128	24	16	32
Antifungal metabolite	0.5–32	16	16	32

^a,b^ 50% and 90%, MICs at which 50 and 90% of isolates are inhibited, respectively; ^c^ in calculation of the median values, MICs of >64 μg/mL were classed as 128 μg/mL.

**Table 4:** ^1^H NMR and ^13^C NMR data of the pure metabolite in ppm (multiplicity, *J* in Hz) ^a^.

Position	δ_H_ (MeOD, 400 MHz, *J* in Hz)	δ_C_ (MeOD, 100 MHz)
1	-	132.1 (C)
2	7.54 (m)	125.9 (CH)
3	7.64 (m)	126.6 (CH)
4	8.2 (m)	123.4 (CH)
5	-	134 (C)
6	7.54 (m)	126.1 (CH)
7	7.93 (m)	128.7 (CH)
8	7.93 (m)	129.7 (CH)
9	7.64 (m)	129.6 (CH)
10	-	131.9 (C)
11	4.4 (s)	56.8 (C)
12	3.6 (d, 7)	58.2 (CH_2_)
13	5.9 (d, 15.9)	118.2 (CH)
14	6.1 (m)	131.7 (CH)
15	-	76.6 (C)
16	-	100.1 (C)
17	-	27.6 (C)
18–20	1.23 (s)	29.9 (CH_3_)
21 (N-CH_3_)	2.52 (s)	39.9 (CH_3_)

^a^ Assignments were done based on COSY, HSQC and HMBC experiments.

## Data Availability

All data are included within the manuscript and supplementary file.

## Supplementary Materials

The following are available online. Table S1: Screening for the antifungal activity of the metabolites produced by isolates S6, F1 and F2, Table S2: The yield of different solvent extracts obtained from *Lysinibacillus* isolate and the corresponding inhibition zones against *C. albicans* and *A. niger*, Table S3: Distribution of MIC for fluconazole, miconazole, lamisil and the antifungal metabolite against six isolates of *C. albicans*, Figure S1: Dual-culture technique showing inhibitory effect of *Lysinibacillus* (S6) against *A. niger*. Left plate shows clear zone between the edges of fungal mycelia and bacterial colonies. Right plate Control plate without tested bacteria, Figure S2: Bioautography method used to determine the antifungal activity of the pooled fractions obtained from column chromatography against *C. albicans*. Left plate highlights the zone of inhibition caused by the fractions with the most potent antifungal activity. Right plate shows no inhibitory effect of purified fractions, Figure S3: The effect of different (a) temperatures, (b) pH, (c) detergents, and enzymes on the stability of the purified antifungal metabolite of *Lysinibacillus* isolate, Figure S4: (a) UV and (b) FTIR spectra of the purified antifungal compound of *Lysinibacillus* isolate, Figure S5 (a) ^1^HNMR and (b) ^13^C NMR spectra of the purified antifungal compound of *Lysinibacillus* isolate, Figure S6: 2D NMR spectra of the purified antifungal compound of *Lysinibacillus* isolate (A) HMBC spectrum, (B) HSQC spectrum and (C) COSY spectrum.

## Sample Availability

Samples of the compounds of this study are available from the authors.

## References

[1] Fuller J., Dingle T.C., Bull A., Shokoples S., Laverdière M., Baxter M.R., Adam H.J., Karlowsky J.A., Zhanel G.G. (2019). Species distribution and antifungal susceptibility of invasive *Candida* isolates from Canadian hospitals: Results of the CANWARD 2011–16 study. J. Antimicrob. Chemother..

[2] Shishodia S.K., Tiwari S., Shankar J. (2019). Resistance mechanism and proteins in *Aspergillus* species against antifungal agents. Mycology.

[3] Pérez-Cantero A., López-Fernández L., Guarro J., Capilla J. (2020). Azole resistance mechanisms in Aspergillus: Update and recent advances. Int. J. Antimicrob. Agents.

[4] Pfavayi L.T., Denning D.W., Baker S., Sibanda E.N., Mutapi F. (2021). Determining the burden of fungal infections in Zimbabwe. Sci. Rep..

[5] Lass-Flörl C., Samardzic E., Knoll M. (2021). Serology anno 2021–fungal infections: From invasive to chronic. Clin. Microbiol. Infect..

[6] Lamoth F., Lockhart S.R., Berkow E.L., Calandra T. (2018). Changes in the epidemiological landscape of invasive candidiasis. J. Antimicrob. Chemother..

[7] Schmidt-Westhausen A.M., Priepke F., Bergmann F.J., Reichart P.A. (2000). Decline in the rate of oral opportunistic infections following introduction of highly active antiretroviral therapy. J. Oral Pathol. Med..

[8] Oliscovicz N., Pomarico L., de Araújo Castro G.F., Souza I.P. (2015). Effect of highly active antiretroviral therapy use on oral manifestations in pediatric patients infected with HIV. Indian J. Dent. Res..

[9] Quindos G., Gil-Alonso S., Marcos-Arias C., Sevillano E., Mateo E., Jauregizar N., Eraso E. (2019). Therapeutic tools for oral candidiasis: Current and new antifungal drugs. Med. Oral Patol. Oral Cir. Bucal.

[10] Bod P., Terdy L., Trischler F., Fekecs E., Demeter M., Lauko A., Domany G., Komlosi G.S., Varga K. (2004). Process for Preparing a Substituted Allylamine Derivative and the Salts Thereof. Google Patents US20030028032A1. https://patents.google.com/patent/US20030028032A1/en.

[11] Stuetz A., Petranyi G. (1984). Synthesis and antifungal activity of (E)-N-(6,6-dimethyl-2-hepten-4-ynyl)-N-methyl-1-naphthalenemethanamine (SF 86-327) and related allylamine derivatives with enhanced oral activity. J. Med. Chem..

[12] Fuglseth E., Otterholt E., Høgmoen H., Sundby E., Charnock C., Hoff B.H. (2009). Chiral derivatives of Butenafine and Terbinafine: Synthesis and antifungal activity. Tetrahedron.

[13] Gupta B., Ravindra Babu B., Gyanda K., Panda S.S., Jain S.C. (2014). Stereoselective methodology for the synthesis of an antifungal allylamine: Terbinafine. Lett. Org. Chem..

[14] Hergert T., Mátravölgyi B., Örkényi R., Éles J., Faigl F. (2021). Multistep batch-flow hybrid synthesis of a terbinafine precursor. J. Flow Chem..

[15] Da Matta D., Souza A., Colombo A. (2017). Revisiting species distribution and antifungal susceptibility of *Candida* bloodstream isolates from Latin American medical centers. J. Fungi.

[16] Shah A.M., Rasool S., Majeed A., Mushtaq S., Khan M.H., Hussain A., Shah A., Hassan Q.P. (2017). Reappraisal of actinomycetes for novel bioactive metabolites. Ann. Phytomed..

[17] Monciardini P., Iorio M., Maffioli S., Sosio M., Donadio S. (2014). Discovering new bioactive molecules from microbial sources. Microb. Biotechnol..

[18] Milshteyn A., Schneider J.S., Brady S.F. (2014). Mining the metabiome: Identifying novel natural products from microbial communities. Chem. Biol..

[19] Charlop-Powers Z., Owen J.G., Reddy B.V.B., Ternei M.A., Brady S.F. (2014). Chemical-biogeographic survey of secondary metabolism in soil. Proc. Natl. Acad. Sci. USA.

[20] El-Sayed S.E., Abdelaziz N.A., El-Housseiny G.S., Aboshanab K.M. (2020). Octadecyl 3-(3, 5-di-tert-butyl-4-hydroxyphenyl) propanoate, an antifungal metabolite of *Alcaligenes faecalis* strain MT332429 optimized through response surface methodology. Appl. Microbiol. Biotechnol..

[21] Nam Y.-D., Seo M.-J., Lim S.-I., Lee S.-Y. (2012). Genome sequence of *Lysinibacillus boronitolerans* F1182, isolated from a traditional Korean fermented soybean product. J. Bacteriol..

[22] Ahmed I., Yokota A., Yamazoe A., Fujiwara T. (2007). Proposal of *Lysinibacillus boronitolerans* gen. nov. sp. nov., and transfer of *Bacillus fusiformis* to *Lysinibacillus fusiformis* comb. nov. and *Bacillus sphaericus* to *Lysinibacillus sphaericus* comb. nov. Int. J. Syst. Evol. Microbiol..

[23] Liu X., Ma Z., Zhang J., Yang L. (2017). Antifungal Compounds against Candida Infections from Traditional Chinese Medicine. Biomed. Res. Int..

[24] Yilmaz M., Soran H., Beyatli Y. (2006). Antimicrobial activities of some Bacillus spp. strains isolated from the soil. Microbiol. Res..

[25] Caulier S., Nannan C., Gillis A., Licciardi F., Bragard C., Mahillon J. (2019). Overview of the antimicrobial compounds produced by members of the Bacillus subtilis group. Front. Microbiol..

[26] Jin P., Wang H., Tan Z., Xuan Z., Dahar G.Y., Li Q.X., Miao W., Liu W. (2020). Antifungal mechanism of bacillomycin D from Bacillus velezensis HN-2 against Colletotrichum gloeosporioides Penz. Pestic. Biochem. Physiol..

[27] Xiao J., Guo X., Qiao X., Zhang X., Chen X., Zhang D. (2021). Activity of fengycin and iturin A isolated from Bacillus subtilis Z-14 on Gaeumannomyces graminis var. tritici and soil microbial diversity. Front. Microbiol..

[28] Lei S., Zhao H., Pang B., Qu R., Lian Z., Jiang C., Shao D., Huang Q., Jin M., Shi J. (2019). Capability of iturin from Bacillus subtilis to inhibit Candida albicans in vitro and in vivo. Appl. Microbiol. Biotechnol..

[29] Ypucef-Ali M., Chaouche N.K., Dehimat L., Bataiche I., Mounira K., Cawoy H.E., Thonart P. (2014). Antifungal activity and bioactive compounds produced by Bacillus mojavensis and Bacillus subtilis. Afr. J. Microbiol. Res..

[30] Jiang J., Gao L., Bie X., Lu Z., Liu H., Zhang C., Lu F., Zhao H. (2016). Identification of novel surfactin derivatives from NRPS modification of Bacillus subtilis and its antifungal activity against Fusarium moniliforme. BMC Microbiol..

[31] Sumi C.D., Yang B.W., Yeo I.-C., Hahm Y.T. (2015). Antimicrobial peptides of the genus Bacillus: A new era for antibiotics. Can. J. Microbiol..

[32] Awais M., Shah A.A., Hameed A., Hasan F. (2007). Isolation, identification and optimization of bacitracin produced by *Bacillus* sp.. Pak. J. Bot..

[33] Mikkola R. (2006). Food and Indoor Air Isolated Bacillus Non-Protein Toxins: Structures, Physico-Chemical Properties and Mechanisms of Effects on Eukaryotic Cells. Ph.D. Thesis.

[34] Devi S., Kiesewalter H.T., Kovács R., Frisvad J.C., Weber T., Larsen T.O., Kovács Á.T., Ding L. (2019). Depiction of secondary metabolites and antifungal activity of Bacillus velezensis DTU001. Synth. Syst. Biotechnol..

[35] Wang X., Zhao D., Shen L., Jing C., Zhang C., Meena V. (2018). Application and Mechanisms of *Bacillus subtilis* in Biological Control of Plant Disease. Role of Rhizospheric Microbes in Soil.

[36] Hossain A.S.M., Sil B.C., Iliopoulos F., Lever R., Hadgraft J., Lane M.E. (2019). Preparation, Characterisation, and Topical Delivery of Terbinafine. Pharmaceutics.

[37] Singh M., Kumar A., Singh R., Pandey K.D. (2017). Endophytic bacteria: A new source of bioactive compounds. 3 Biotech.

[38] Mahendran S., Vijayabaskar P., Saravanan S., An K., Shankar T. (2013). Structural characterization and biological activity of exopolysaccharide from *Lysinibacillus fusiformis*. Afr. J. Microbiol. Res..

[39] Naureen Z., Rehman N.U., Hussain H., Hussain J., Gilani S.A., Al Housni S.K., Mabood F., Khan A.L., Farooq S., Abbas G. (2017). Exploring the potentials of *Lysinibacillus sphaericus* ZA9 for plant growth promotion and biocontrol activities against phytopathogenic fungi. Front. Microbiol..

[40] Ahmad V., Iqbal A.N., Haseeb M., Khan M.S. (2014). Antimicrobial potential of bacteriocin producing *Lysinibacillus* jx416856 against foodborne bacterial and fungal pathogens, isolated from fruits and vegetable waste. Anaerobe.

[41] Singh R.K., Kumar D.P., Solanki M.K., Singh P., Srivastva A.K., Kumar S., Kashyap P.L., Saxena A.K., Singhal P.K., Arora D.K. (2013). Optimization of media components for chitinase production by chickpea rhizosphere associated *Lysinibacillus fusiformis* B-CM18. J. Basic Microbiol..

[42] El-Sayed S.E., El-Housseiny G.S., Abdelaziz N.A., El-Ansary M.R., Aboshanab K.M. (2020). Optimized Production of the Allylamine Antifungal “Terbinafine” by *Lysinibacillus* Isolate MK212927 Using Response Surface Methodology. Infect. Drug Resist..

[43] Parthasarathi S., Sathya S., Bupesh G., Manikandan M., Kim C., Manikandan T., Balakrishnan K. (2012). Isolation, Characterization and Extraction of antimicrobial compound from marine actinomycete Streptomyces hygroscopicus BDUS 49. Res. J. Biotechnol..

[44] Thangadurai D., Murthy K., Prasad P., Pullaiah T. (2004). Antimicrobial screening of Decalepis hamiltonii wight and arn.(Asclepiadaceae) root extracts against food-related microorganisms. J. Food Saf..

[45] Chawawisit K., Bhoopong P., Phupong W., Lertcanawanichakul M. (2015). 2,4-Di-tert-butylphenol, the bioactive compound produced by *Streptomyces* sp. KB1. J. Appl. Pharm. Sci..

[46] Motta A., Brandelli A. (2002). Characterization of an antibacterial peptide produced by *Brevibacterium linens*. J. Appl. Microbiol..

[47] Morschhäuser J. (2016). The development of fluconazole resistance in *Candida albicans*—An example of microevolution of a fungal pathogen. J. Microbiol..

[48] Ali G.S., El-Sayed A.S., Patel J.S., Green K.B., Ali M., Brennan M., Norman D. (2016). Ex Vivo Application of Secreted Metabolites Produced by Soil-Inhabiting *Bacillus* spp. Efficiently Controls Foliar Diseases Caused by *Alternaria* spp.. Appl. Environ. Microbiol..

[49] Kumar A., Zarychanski R., Pisipati A., Kumar A., Kethireddy S., Bow E.J. (2018). Fungicidal versus fungistatic therapy of invasive *Candida* infection in non-neutropenic adults: A meta-analysis. Mycology.

[50] Gupta A.K., Drummond-Main C. (2013). Meta-analysis of randomized, controlled trials comparing particular doses of griseofulvin and terbinafine for the treatment of tinea capitis. Pediatr. Dermatol..

[51] Muharram M., Abdel-Kader M. (2014). Taxonomic characterization and chemical study of the antifungal constituents of *Streptomyces* sp. KH-F12. J. Biol. Sci..

[52] Kind T., Fiehn O. (2010). Advances in structure elucidation of small molecules using mass spectrometry. Bioanal. Rev..

[53] Abdel-Rahman S.M., Nahata M.C. (1997). Oral terbinafine: A new antifungal agent. Ann. Pharmacother..

[54] Maxfield L., Preuss C.V., Bermudez R. (2021). Terbinafine.

[55] Elewski B., Tavakkol A. (2005). Safety and tolerability of oral antifungal agents in the treatment of fungal nail disease: A proven reality. Ther. Clin. Risk Manag..

[56] Chubukov V., Mukhopadhyay A., Petzold C.J., Keasling J.D., Martín H.G. (2016). Synthetic and systems biology for microbial production of commodity chemicals. NPJ Syst. Biol. Appl..

[57] Abdel-Kader M.S., Muharram M.M. (2017). New microbial source of the antifungal allylamine “Terbinafine”. Saudi Pharm. J..

[58] Hossain N., Rahman M. (2014). Antagonistic activity of antibiotic producing *Streptomyces* sp. against fish and human pathogenic bacteria. Braz. Arch. Biol. Technol..

[59] Rahman M.A., Islam M.Z., Islam M.A. (2011). Antibacterial activities of actinomycete isolates collected from soils of rajshahi, bangladesh. Biotechnol. Res. Int..

[60] Peela S., Kurada V.B., Terli R. (2005). Studies on antagonistic marine actinomycetes from the Bay of Bengal. World J. Microbiol. Biotechnol..

[61] Tiru M., Muleta D., Bercha G., Adugna G. (2013). Antagonistic effect of rhizobacteria against coffee wilt disease caused by *Gibberella xylarioides*. Asian J. Plant Pathol..

[62] Montealegre J.R., Reyes R., Pérez L.M., Herrera R., Silva P., Besoain X. (2003). Selection of bioantagonistic bacteria to be used in biological control of *Rhizoctonia solani* in tomato. Electron. J. Biotechnnol..

[63] Magaldi S., Mata-Essayag S., Hartung de Capriles C., Perez C., Colella M.T., Olaizola C., Ontiveros Y. (2004). Well diffusion for antifungal susceptibility testing. Int. J. Infect. Dis..

[64] Wiegand I., Hilpert K., Hancock R.E. (2008). Agar and broth dilution methods to determine the minimal inhibitory concentration (MIC) of antimicrobial substances. Nat. Protoc..

[65] Bundale S., Begde D., Nashikkar N., Kadam T., Upadhyay A. (2014). Isolation of aromatic polyketide producing soil *Streptomyces* using combinatorial screening strategies. Open Access Libr. J..

[66] Rojas J.J., Ochoa V.J., Ocampo S.A., Muñoz J.F. (2006). Screening for antimicrobial activity of ten medicinal plants used in Colombian folkloric medicine: A possible alternative in the treatment of non-nosocomial infections. BMC Complement. Altern. Med..

[67] Moreno-Arribas M.V., Polo M.C. (2008). Occurrence of lactic acid bacteria and biogenic amines in biologically aged wines. Food Microbiol..

[68] Kumar S., Stecher G., Li M., Knyaz C., Tamura K. (2018). MEGA X: Molecular Evolutionary Genetics Analysis across Computing Platforms. Mol. Biol. Evol..

[69] Kimura M. (1980). A simple method for estimating evolutionary rates of base substitutions through comparative studies of nucleotide sequences. J. Mol. Evol..

[70] Bhosale H., Kadam T., Mirajgave R., Holkar S. (2018). Optimization and characterization of antifungal metabolite from a soil actinomycete *Streptomyces indiaensis* SRT1. Indian J. Biotechnol..

[71] Kumar P.S., Duraipandiyan V., Ignacimuthu S. (2014). Isolation, screening and partial purification of antimicrobial antibiotics from soil Streptomyces sp. SCA 7. Kaohsiung J. Med. Sci..

[72] Balouiri M., Bouhdid S., Harki E., Sadiki M., Ouedrhiri W., Ibnsouda S.K. (2015). Antifungal activity of *Bacillus* spp. isolated from *Calotropis procera* AIT. Rhizosphere against *Candida albicans*. Asian J. Pham. Clin. Res..

[73] Augustine S., Bhavsar S., Kapadnis B. (2005). A non-polyene antifungal antibiotic from *Streptomyces albidoflavus* PU 23. J. Biosci..

[74] Munimbazi C., Bullerman L. (1998). Isolation and partial characterization of antifungal metabolites of *Bacillus pumilus*. J. Appl. Microbiol..

[75] Shahidi Bonjar G., Rashid Farrokhi P., Aghighi S., Shahidi Bonjar L., Aghelizadeh A. (2005). Antifungal characterization of actinomycetes isolated from Kerman, Iran and their future prospects in biological control strategies in greenhouse and field conditions. Plant Pathol. J..

[76] Navi S., Rajasab A., Yang X. (2016). In Vitro evaluation of commercial fungicides against some of the major soil borne pathogens of soybean. J. Plant Pathol. Microbiol..

[77] CLSI (2008). Reference Method for Broth Dilution Antifungal Susceptibility Testing of Yeasts, Approved Standard.

