# Supplementary material for: Lysinibacillus Isolate MK212927: A Natural Producer of Allylamine Antifungal ‘Terbinafine’
Source: Molecules. 2021 Dec 29;27(1):201. doi: 10.3390/molecules27010201 (PMC8746802; doi:10.3390/molecules27010201)
Supplement: Supplementary file 1 [file molecules-27-00201-s001.zip › molecules-1518158-supplementary.pdf]

Table S1 Screening for the antifungal activity of the metabolites produced by isolates S6, F1and F2

**Isolate**	**Mean inhibition zones against *C. albicans* ATCC 10231 in Agar well diffusion method (mm) ±SD**	**Mean inhibition zones against *A. niger* (mm) ± SD in dual culture method (mm) ±SD**	**Radial mycelial growth of *A. niger* in the presence of the tested isolate in dual culture method (cm)**	**Radial mycelial growth inhibition (%)***
S6	19.3 ±0.58	12.67 ±0.58	1.25	83.33
F1	12.6 ±0.58	8 ± 0.36	2.9	61.3
F2	15 ± 0.25	10 ±0 .5	2.5	66.66

* Radial mycelial growth *of A. niger* without the tested isolate = 7.5 cm.. SD (standard deviation)

Table S2 The yield of different solvent extracts obtained from *Lysinibacillus* isolate and the corresponding inhibition zones against *C. albicans* and *A. niger*

Solvent	Yield of extraction (g/200ml)	Mean inhibition zones (mm) ±SD	
		C. albicans	*A. niger*
Ethyl acetate	1.07	26.6 ±0.58	24.3 ±0.58
n-hexane	0.91	21.3 ±0.5	20.3 ±0.36
Chloroform	0.68	18.3 ±0.45	20.3 ±0.36
Diethyl ether	0.63	19.67 ±0.36	18.3 ±0.58
n-butanol (n-butyl alcohol)	0.40	15 ±0.25	12.3 ±0.25
Dichloromethane	0.35	14.3 ±0.58	13 ±0.23
Ethanol	0.12	11 ±0.23	11.3 ±0.50
Methanol	-	-	-

SD (standard deviation)

Table S3 Distribution of MIC for fluconazole, miconazole, Lamisil and the antifungal metabolite against six isolates of *C. albicans*

Antifungal agents	MIC (μg/ml)										
	**0.125**	**0.25**	**0.5**	**1**	**2**	**4**	**8**	**16**	**32**	**64**	**128**
Fluconazole	-	-	-	1	-	-	1	1	1	-	2
Miconazole	-	-	-	-	1	-	-	-	2	2	1
Lamisil	-	-	-	1	-	1	-	1	2	-	1
Antifungal metabolite	-	-	1	-	-	-	1	2	2	-	-

Supplementary Figures:

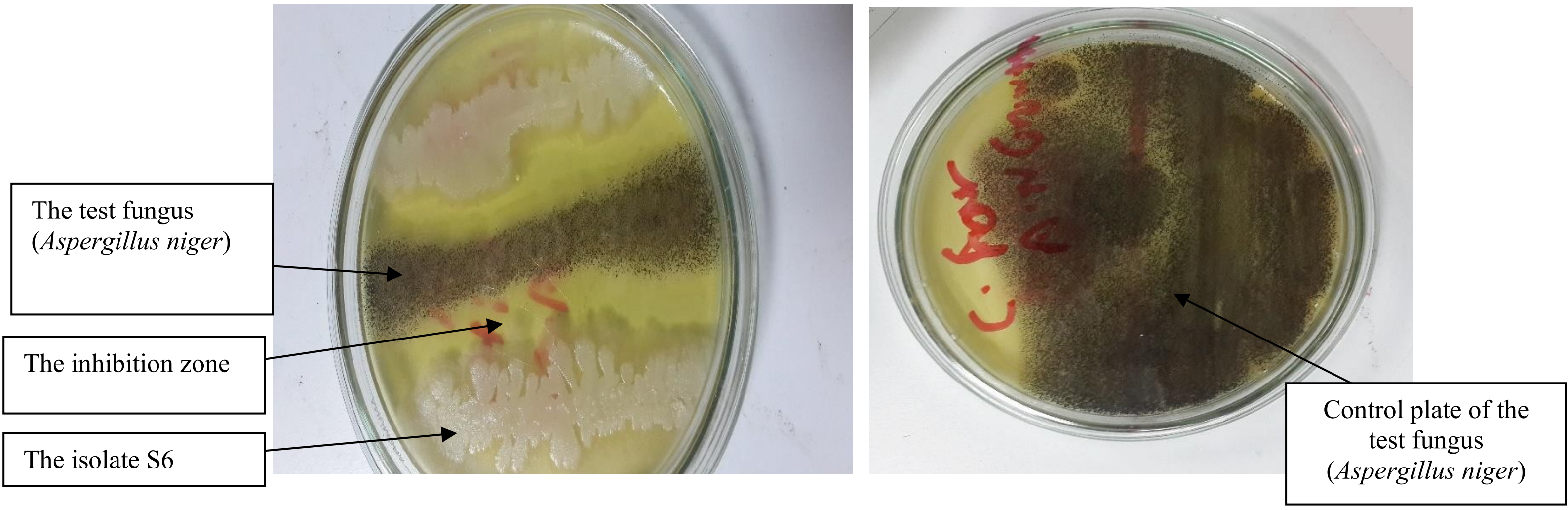

Figure S1 Dual culture technique showing inhibitory effect of *Lysinibacillus* (S6) against *A. niger*. *Left plate* shows clear zone between the edges of fungal mycelia and bacterial colonies. *Right plate* Control plate without tested bacteria

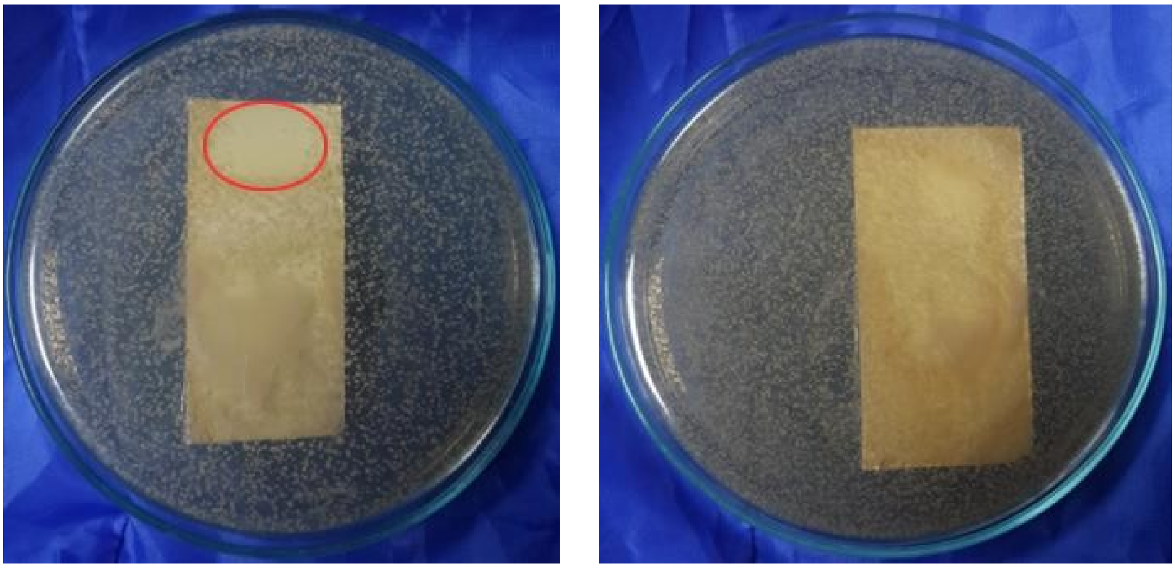

Figure S2 Bioautography method used to determine the antifungal activity of the pooled fractions obtained fromcolumn chromatography against *C. albicans. Left plate* highlights the zone of inhibition caused by the fractionswith the most potent antifungal activity. *Right plate* shows no inhibitory effect of purified fractions

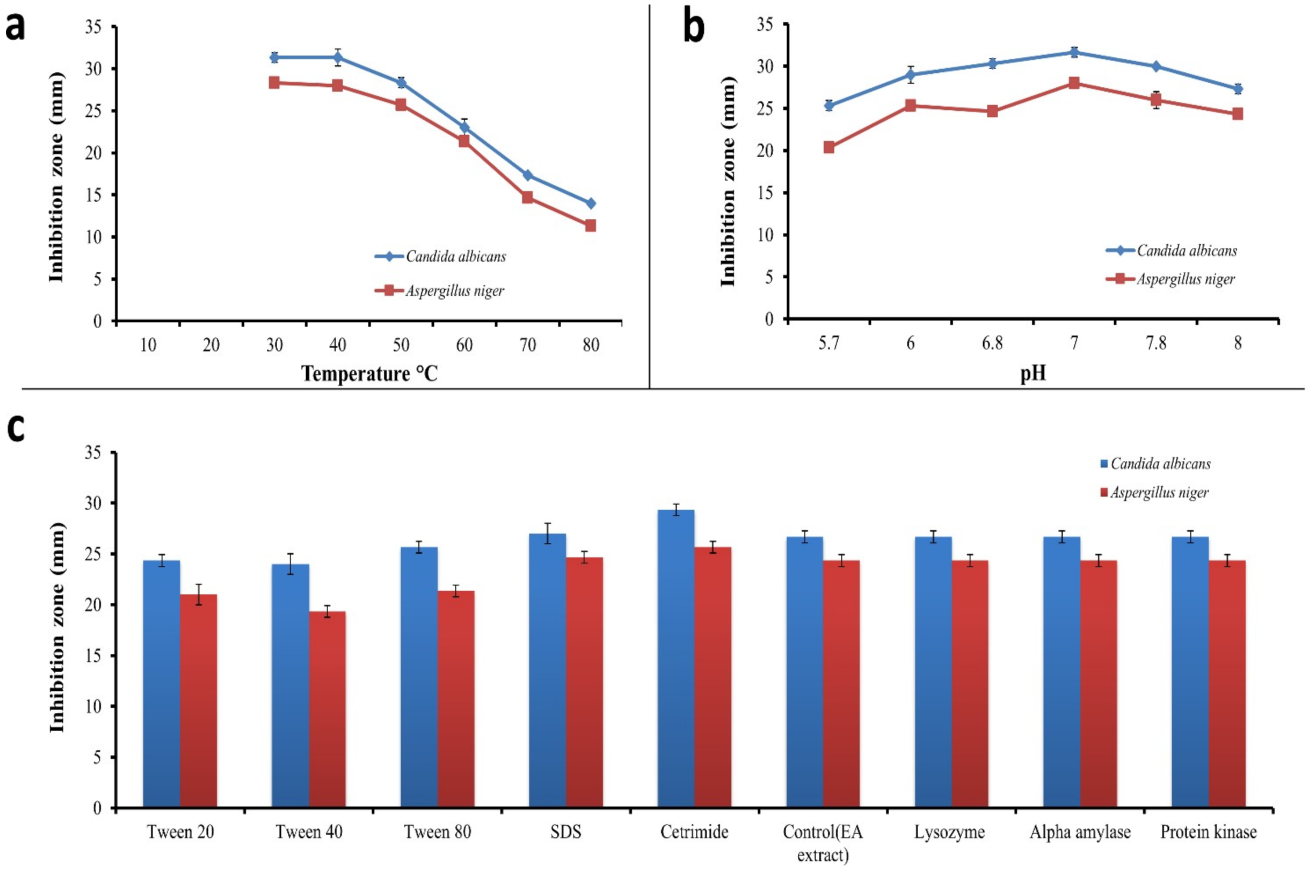

Figure S3 The effect of different (a) Temperatures, (b) pH, (c) Detergents, and enzymes on the stability of the purifiedantifungal metabolite of *Lysinibacillus* isolate

a

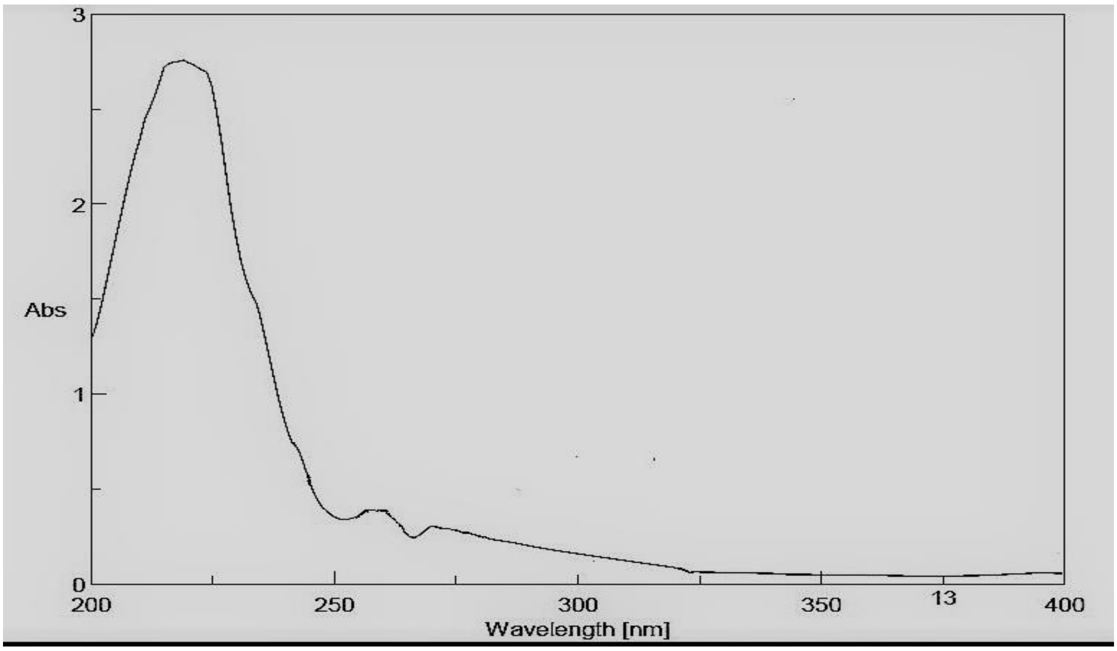

-UV spectra of Terbinafine metabolite

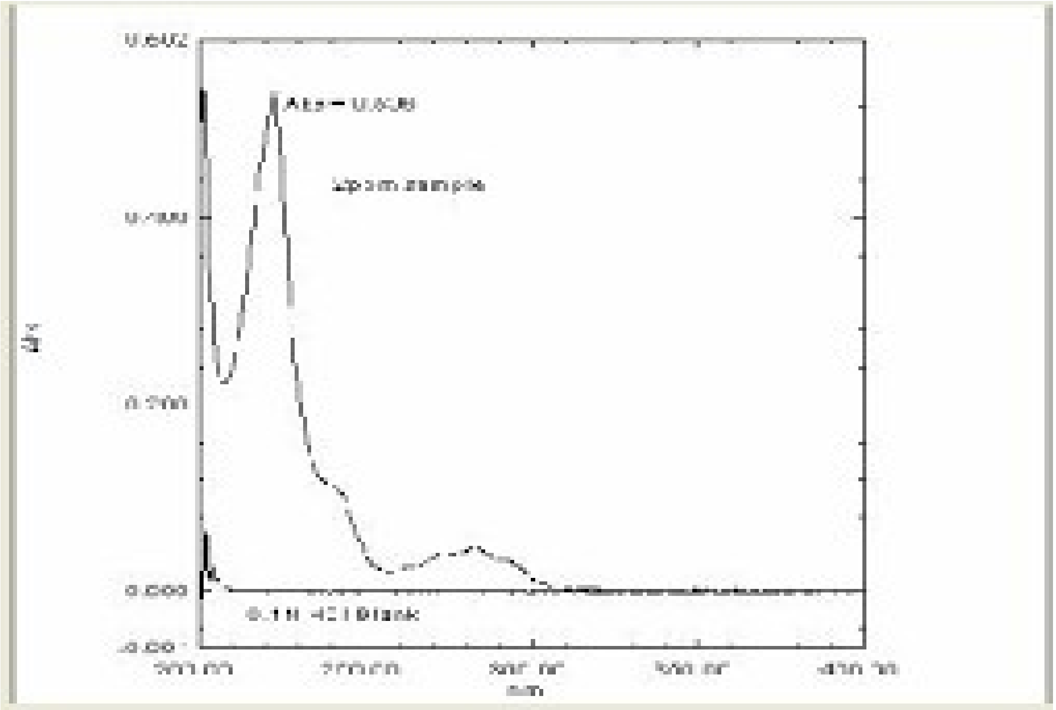

- UV Spectra of Standard Terbinafine (https://sphinxsai.com/2013/JulySept13/chPDF/CT=86(2645-2655)JS13.pdf)

B

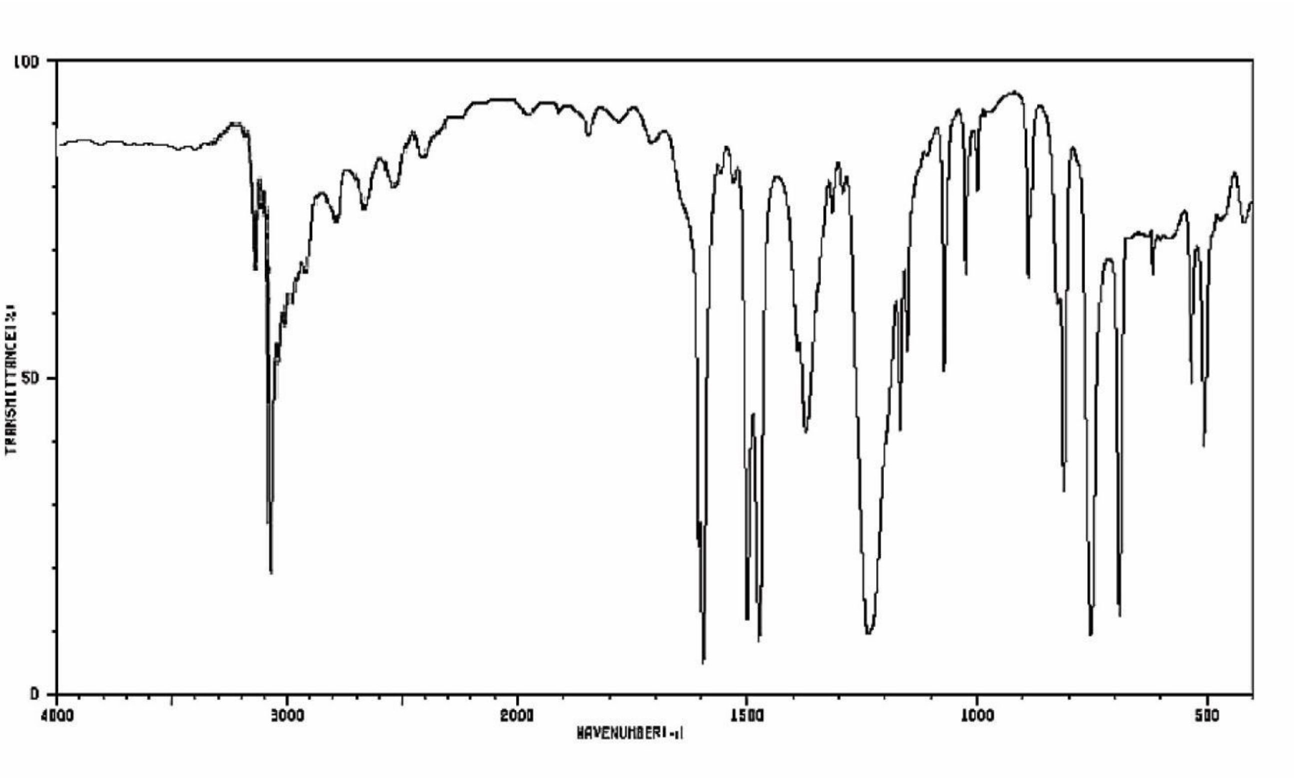

- FTIR spectra of terbinafine metabolite

Figure S4 (a) UV and (b) FTIR spectra of the purified antifungal compound of *Lysinibacillus* isolate. and it was compa
the standard terbinafine found in the Reaxys database (**https://0812939fu-1105-y-https-www-reaxys-com.mplbci.ekb.eg/#/results/substances/0/RX001_5178259681407157012/UlgwMDE9UyNIMDA2PUMjSDAwNz1S/092e-4706-994b-82a6a743172d/1/desc/IDE.NUMREF///**)

a

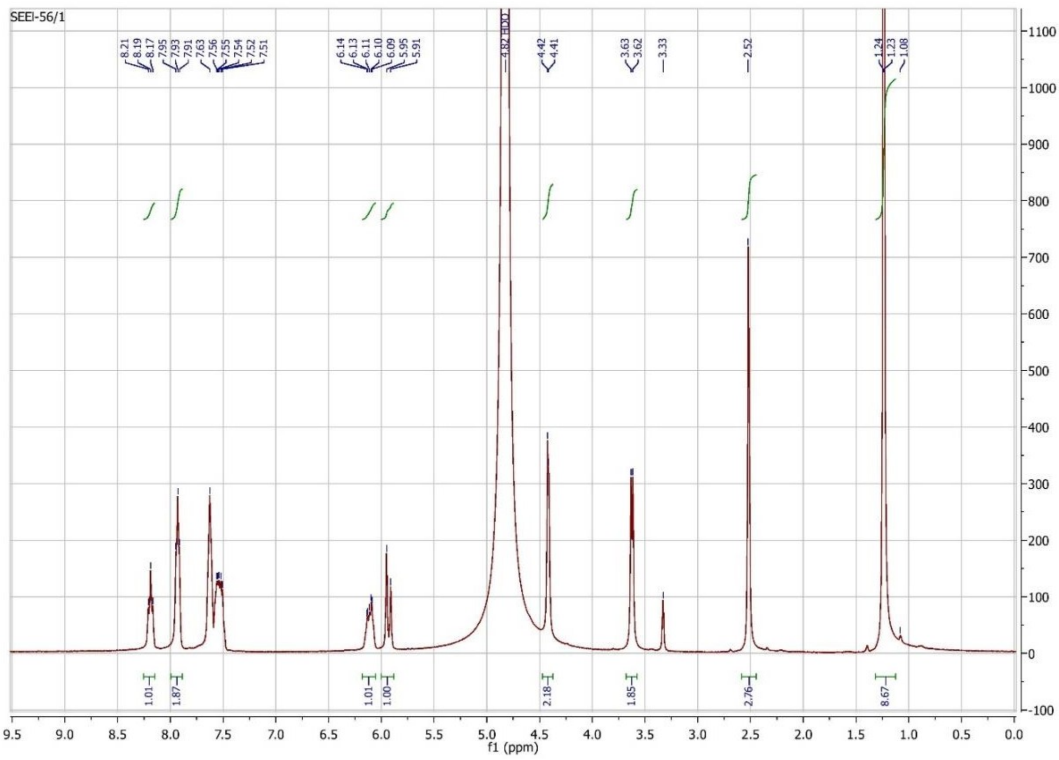

b

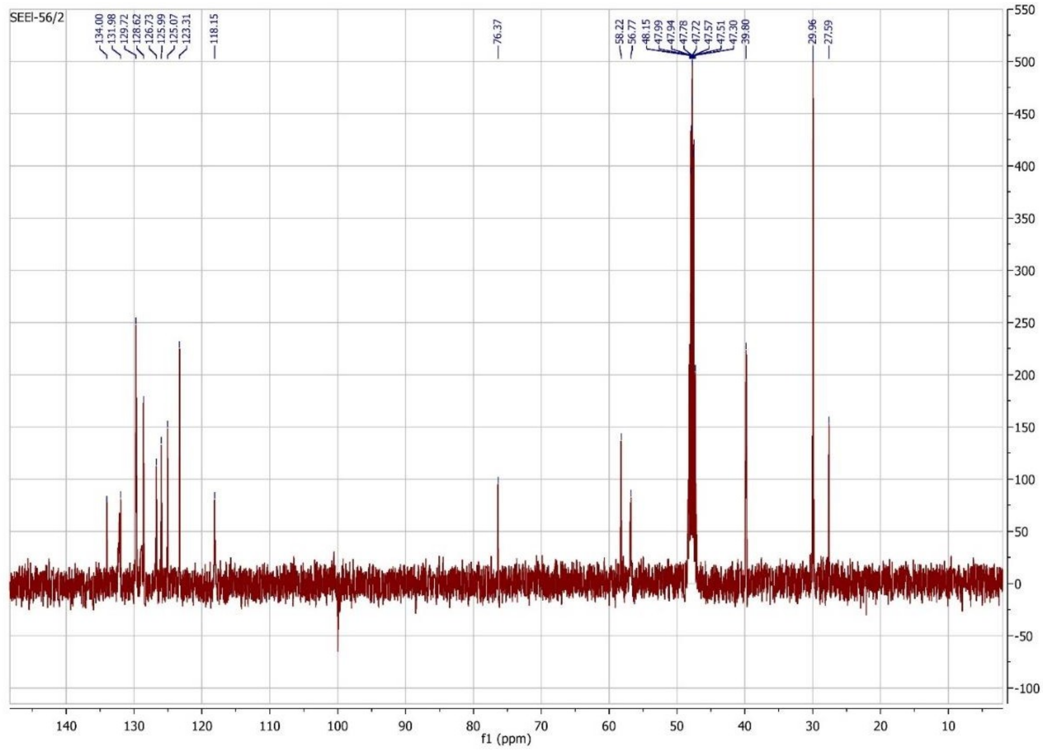

Figure S5 (a) ^{1}HNMR and (b) ^{13}C NMR spectra of the purified antifungal compound of *Lysinibacillus* isolate

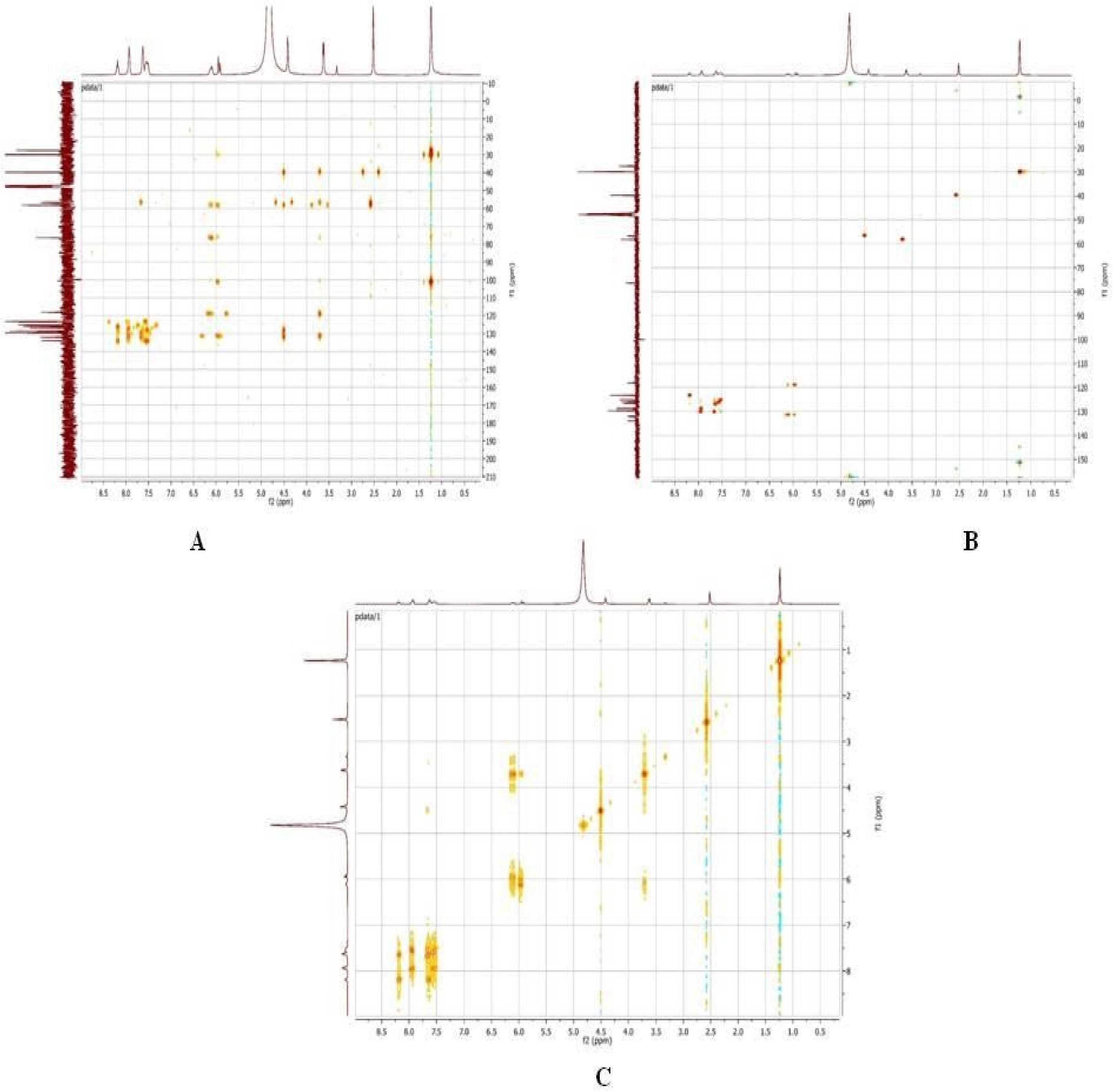

Figure S6 2D NMR spectra of the purified antifungal compound of *Lysinibacillus* isolate (A) HMBC spectrum, (B) HSQC spectrum and (C) COSY spectrum
